# Supplementary material for: Untargeted metabolomics for the early detection of preeclampsia: A systematic review of human studies
Source: PLoS One. 2026 Mar 30;21(3):e0339292. doi: 10.1371/journal.pone.0339292 (PMC13035155; doi:10.1371/journal.pone.0339292)
Supplement: S1 Appendix — (DOCX) [file pone.0339292.s001.docx]

**S1 Appendix. Review Objectives and Research Questions**

**Objective**: To synthesize current evidence on metabolomics-based biomarkers associated with preeclampsia, with a focus on identifying promising candidates for early prediction or diagnosis and potential clinical translation.

To address this aim, the present review is guided by the following research questions:

- What metabolomic alterations have been identified in patients with preeclampsia compared to healthy pregnancies?
  - Are these findings consistent across different biological samples?
  - Are specific metabolic pathways or biomarkers repeatedly implicated?
- What types of metabolomic techniques and analytical platforms have been used in the study of preeclampsia?
- What are the potential roles of metabolomics in understanding the pathophysiology of preeclampsia?
- What are the main limitations among the available studies?
- What gaps exist in the current literature, and what directions should future research take to improve the clinical utility of metabolomics in preeclampsia?
