# Supplementary material for: Untargeted metabolomics for the early detection of preeclampsia: A systematic review of human studies
Source: PLoS One. 2026 Mar 30;21(3):e0339292. doi: 10.1371/journal.pone.0339292 (PMC13035155; doi:10.1371/journal.pone.0339292)
Supplement: S2 Appendix — (DOCX) [file pone.0339292.s002.docx]

**S2 Appendix. PICOS Criteria for Study Selection**

**Population (P):**

Pregnant individuals at risk of or diagnosed with preeclampsia.

- Single or multiple pregnancies
- Women in any trimester of pregnancy
- Pregnant women with clinical risk factors for preeclampsia
- Studies including participants who developed preeclampsia at any stage (early-onset or late-onset)

**Intervention (I):**

Application of metabolomic profiling or metabolite analysis aimed at identifying biomarkers associated with preeclampsia.

- Metabolomic studies using mass spectrometry (MS), nuclear magnetic resonance (NMR), liquid or gas chromatography, or other validated metabolomic techniques
- Analysis of different biological matrixes

**Comparator (C):**

Healthy pregnant individuals without preeclampsia or related hypertensive disorders, used as controls.

**Outcomes (O):**

Identification, characterization, and/or validation of metabolomic biomarkers associated with preeclampsia.

- Metabolites or metabolic pathways that distinguish preeclamptic from normotensive pregnancies
- Associations between specific metabolites and clinical severity, onset timing, or pathophysiological mechanisms of preeclampsia

**Study Design (S):**

Cohort studies, case-cohort studies, case-control studies, validation studies, prospective control studies, and translational studies.
