# Supplementary material for: Untargeted metabolomics for the early detection of preeclampsia: A systematic review of human studies
Source: PLoS One. 2026 Mar 30;21(3):e0339292. doi: 10.1371/journal.pone.0339292 (PMC13035155; doi:10.1371/journal.pone.0339292)
Supplement: S1 Table — (DOCX) [file pone.0339292.s003.docx]

**S3 Table. Critical appraisal for the selected studies**

| **Study** | **Sample size sufficient for robust metabolomic profiling** | **Appropiate inclusion/exclusion criteria** | **Measurement of biomarkers** | **External validity** | **Overall** |
| --- | --- | --- | --- | --- | --- |
| Austdal, M. et al 2015a | Low | Low | Unclear | Unclear | Low |
| de Almeida, L.G.N. et al. 2022 | High | Unclear | Unclear | High | Unclear |
| Austdal, M. et al. 2019 | Low | Low | High | High | Unclear |
| Kenny, L.C. et al. 2010 | Low | High | High | Low | Unclear |
| Liu, X. et al. 2024 | Low | Low | Unclear | Unclear | Low |
| Austdal, M. et al 2015b | Low | High | Unclear | Low | Unclear |
| Sovio, U. et al. 2020 | High | Unclear | High | Low | Low |
| Bahado-Singh, R.O. et al. 2015 | Low | Unclear | High | Low | Low |
| Sander, K.N. et al. 2019 | Low | Low | Unclear | High | Unclear |
| Harville, E.W. et al. 2021 | High | Unclear | High | High | Unclear |
| Ferranti, E.P. et al. 2020 | High | Unclear | Low | Low | Low |
| Odibo, A.O. et al. 2011 | Low | Unclear | Low | High | Unclear |
