# Supplementary material for: Untargeted metabolomics for the early detection of preeclampsia: A systematic review of human studies
Source: PLoS One. 2026 Mar 30;21(3):e0339292. doi: 10.1371/journal.pone.0339292 (PMC13035155; doi:10.1371/journal.pone.0339292)
Supplement: S1 Fig — Risk of bias assessment across the included studies using the CADIMA criteria. The figure summarizes the evaluation of each study based on four domains: D1 (sample size sufficiency for metabolomic profiling), D2 (clarity of inclusion/exclusion criteria), D3 (biomarker measurement), and D4 (external validity). Judgements are categorized as low risk (+), unclear risk (–), or high risk (x). Overall risk of bias reflects the combined judgement across all four domains. The visualization was generated using the robvis tool [63]. (DOCX) [file pone.0339292.s004.docx]

**S4 Appendix. Risk of bias assessment of the included studies.**


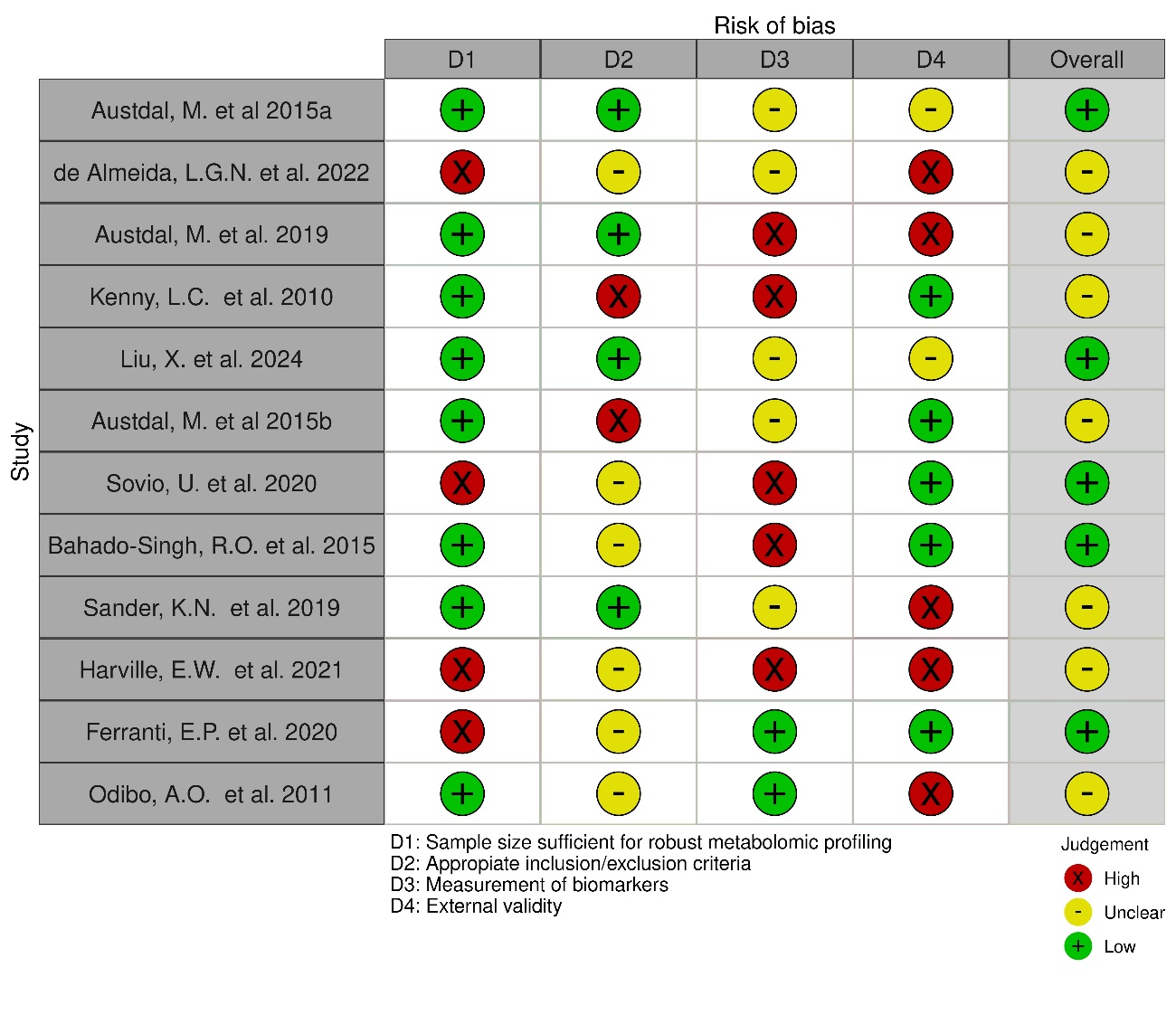
**Figure 1**. Risk of bias assessment across the included studies using the CADIMA criteria. The figure summarizes the evaluation of each study based on four domains: D1 (sample size sufficiency for metabolomic profiling), D2 (clarity of inclusion/exclusion criteria), D3 (biomarker measurement), and D4 (external validity). Judgements are categorized as low risk (+), unclear risk (–), or high risk (x). Overall risk of bias reflects the combined judgement across all four domains. The visualization was generated using the robvis tool [1].

1. McGuinness LA, Higgins JPT. Risk-of-bias VISualization (robvis): An R package and Shiny web app for visualizing risk-of-bias assessments. Res Synth Methods. 2020;n/a. doi:10.1002/jrsm.1411
