# Supplementary material for: Untargeted metabolomics for the early detection of preeclampsia: A systematic review of human studies
Source: PLoS One. 2026 Mar 30;21(3):e0339292. doi: 10.1371/journal.pone.0339292 (PMC13035155; doi:10.1371/journal.pone.0339292)
Supplement: S2 Table — (DOCX) [file pone.0339292.s005.docx]

**S5 Table. Excluded studies at title/abstract screening**

| **Author** | **Title** | **Principal r.eason for exclusion** |
| --- | --- | --- |
| Elhakeem, A. et al. 2023 | Effect of common pregnancy and perinatal complications on offspring metabolic traits across the life course: a multi-cohort study. | wrong outcome |
| Hodgman, C. et al. 2022 | Coenzyme A Restriction as a Factor Underlying Pre-Eclampsia with Polycystic Ovary Syndrome as a Risk Factor. | wrong outcome |
| McClements, L. et al. 2022 | Impact of reduced uterine perfusion pressure model of preeclampsia on metabolism of placenta, maternal and fetal hearts. | wrong population |
| Kivelä, J. et al. 2021 | Longitudinal Metabolic Profiling of Maternal Obesity, Gestational Diabetes, and Hypertensive Pregnancy Disorders. | wrong outcome |
| Langston-Cox, A.G. et al. 2021 | Sulforaphane Bioavailability and Effects on Blood Pressure in Women with Pregnancy Hypertension. | wrong outcome |
| Wang, G. et al. 2020 | Contribution of placental 11β-HSD2 to the pathogenesis of preeclampsia. | wrong outcome |
| Khashan, Ali S. et al. 2019 | Preeclampsia and risk of end stage kidney disease: A Swedish nationwide cohort study. | wrong outcome |
| Liu, C. et al. 2017 | Metabolomic Approach in Probing Drug Candidates. | wrong outcome |
| Ornaghi, S. et al. 2015 | Thrombosis during pregnancy: Risks, prevention, and treatment for mother and fetus--harvesting the power of omic technology, biomarkers and in vitro or in vivo models to facilitate the treatment of thrombosis. | wrong outcome |
| McCowan, L. et al. 2013 | Clinical prediction in early pregnancy of infants small for gestational age by customised birthweight centiles: findings from a healthy nulliparous cohort. | wrong outcome |
| Delles, C. et al. 2012 | Proteomics in hypertension and other cardiovascular diseases. | wrong outcome |
| Girchenko, P. et al. 2024 | Associations of polymetabolic risk of high maternal pre-pregnancy body mass index with pregnancy complications, birth outcomes, and early childhood neurodevelopment: findings from two pregnancy cohorts. | wrong outcome |
| Pels, A. et al. 2023 | Interventions affecting the nitric oxide pathway versus placebo or no therapy for fetal growth restriction in pregnancy. | wrong outcome |
| Zhu, A. et al. 2023 | Commentary: magnetic resonance spectroscopy and liquid chromatography-mass spectrometry metabolomics study may differentiate pre-eclampsia from gestational hypertension. | wrong outcome |
| Yang, Y. et al. 2023 | Prediction of hypertensive disorders of pregnancy using metabolomics: there is a long way to go. | wrong outcome |
| Jin, J. et al. 2022 | Gut Dysbiosis Promotes Preeclampsia by Regulating Macrophages and Trophoblasts. | wrong outcome |
| Aalami-Harandi, R. et al. 2015 | The favorable effects of garlic intake on metabolic profiles, hs-CRP, biomarkers of oxidative stress and pregnancy outcomes in pregnant women at risk for pre-eclampsia: randomized, double-blind, placebo-controlled trial | wrong outcome |
| Mesdaghinia, E. et al. 2023 | The Effect of Selenium Supplementation on Clinical Outcomes, Metabolic Profiles, and Pulsatility Index of the Uterine Artery in High-Risk Mothers in Terms of Preeclampsia Screening with Quadruple Test: a Randomized, Double-Blind, Placebo-Controlled Clinical Trial: selenium and preeclampsia | wrong outcome |
| Rezvan Aalami-Harandi, M.K. & Zatollah Asemi 2014 | Statement of Retraction: the favorable effects of garlic intake on metabolic profiles, hs-CRP, biomarkers of oxidative stress and pregnancy outcomes in pregnant women at risk for pre-eclampsia: randomized, double-blind, placebo-controlled trial | wrong outcome |
| Meek, C.L. Et al. 2021 | Use of noncarbohydrate fuels is associated with maternofetal complications in type 1 diabetes pregnancy: metabolomics analysis of the conceptt trial | wrong outcome |
| Huhtala, M.S. | Cord serum metabolome and birth weight in patients with gestational diabetes treated with metformin, insulin, or diet alone | wrong outcome |
| Erchick, D.J. et al. 2023 | Supplementation with fortified balanced energy–protein during pregnancy and lactation and its effects on birth outcomes and infant growth in southern Nepal: protocol of a 2×2 factorial randomised trial | wrong outcome |
| Weiss, S.T. et al. 2014 | Prenatal vitamin D supplementation to prevent childhood asthma: 15-year results from the Vitamin D Antenatal Asthma Reduction Trial (VDAART) | wrong outcome |
| Li, X.Q. et al. 2021 | Effective Aspirin Treatment of Women at Risk for Preeclampsia Delays the Metabolic Clock of Gestation | wrong outcome |
| Heazell, A.E.P. et al. 2011 | The effects of oxygen on normal and pre-eclamptic placental tissue - insights from metabolomics | wrong study design |
| Mohaupt, M. et al. 2007 | Molecular aspects of preeclampsia | wrong outcome |
| Kawasaki, K. et al. 2019 | Metabolomic Profiles of Placenta in Preeclampsia Antioxidant Effect of Magnesium Sulfate on Trophoblasts in Early-Onset Preeclampsia | wrong outcome |
| Diaz, S.O. et al. 2013 | Following Healthy Pregnancy by Nuclear Magnetic Resonance (NMR) Metabolic Profiling of Human Urine | wrong population |
| Benny, P.A. et al. 2020 | A review of omics approaches to study preeclampsia | wrong outcome |
| Mohammad, S. et al. 2021 | Metabolomics to understand placental biology: Where are we now? | wrong outcome |
| Bankole, T. et al. 2022 | Dietary Impacts on Gestational Diabetes: Connection between Gut Microbiome and Epigenetic Mechanisms | wrong outcome |
| Fattuoni, C. et al. 2017 | Preliminary metabolomics analysis of placenta in maternal obesity | wrong outcome |
| He, B. et al. 2021 | The maternal blood lipidome is indicative of the pathogenesis of severe preeclampsia | wrong outcome |
| Francis, E.C. et al. 2024 | Maternal Serum Metabolomics in Mid-Pregnancy Identifies Lipid Pathways as a Key Link to Offspring Obesity in Early Childhood | wrong population |
| Taylor, K. et al. 2019 | Differences in Pregnancy Metabolic Profiles and Their Determinants between White European and South Asian Women: Findings from the Born in Bradford Cohort | wrong outcome |
| Troisi, J. et al. 2023 | Placental Metabolomics of Fetal Growth Restriction | wrong outcome |
| Diaz, S.O. et al. 2011 | Metabolic Biomarkers of Prenatal Disorders: An Exploratory NMR Metabonomics Study of Second Trimester Maternal Urine and Blood Plasma | wrong population |
| Ma, L. et al. 2017 | Urinary metabolomic analysis of intrahepatic cholestasis of pregnancy based on high performance liquid chromatography/mass spectrometry | wrong outcome |
| Yu, Z.E. et al. 2023 | Extracellular Vesicles Derived from Human Umbilical Cord MSC Improve Vascular Endothelial Function in In Vitro and In Vivo Models of Preeclampsia through Activating Arginine Metabolism | wrong outcome |
| Gilley, S.P. et al. 2020 | Longitudinal Changes of One-Carbon Metabolites and Amino Acid Concentrations during Pregnancy in the Women First Maternal Nutrition Trial | wrong outcome |
| Sadovsky, Y. et al. 2020 | Advancing human health in the decade ahead: pregnancy as a key window for discovery A Burroughs Wellcome Fund Pregnancy Think Tank | wrong outcome |
| Saini, N. et al. 2021 | Global metabolomic profiling reveals hepatic biosignatures that reflect the unique metabolic needs of late-term mother and fetus | wrong outcome |
| Chen, J.D. et al. 2020 | Metabolic disparities of different oxidative stress-inducing conditions in HTR8/SVneo cells | wrong outcome |
| Tarca, A.L. et al. 2021 | Maternal whole blood mRNA signatures identify women at risk of early preeclampsia: a longitudinal study | wrong outcome |
| Harrington, J.M. et al. 2016 | Validation of a Metallomics Analysis of Placenta Tissue by Inductively-Coupled Plasma Mass Spectrometry | wrong outcome |
| Sulek, K. et al. 2014 | Hair Metabolomics: Identification of Fetal Compromise Provides Proof of Concept for Biomarker Discovery | wrong outcome |
| Barak, O. et al. 2023 | Integrated unbiased multiomics defines disease-independent placental clusters in common obstetrical syndromes | wrong outcome |
| Kharb, S. et al. 2023 | Multi-omics and machine learning for the prevention and management of female reproductive health | wrong outcome |
| Pantham, P. et al. 2015 | Antiphospholipid Antibodies Alter Cell-Death-Regulating Lipid Metabolites in First and Third Trimester Human Placentae | wrong outcome |
| Lenaerts, C. et al. 2018 | Revealing of endogenous Marinobufagin by an ultra-specific and sensitive UHPLC-MS/MS assay in pregnant women | wrong outcome |
| Chen, Q. et al. 2020 | Metabolic reprogramming by Zika virus provokes inflammation in human placenta | wrong outcome |
| ClinicalTrials.gov ID: NCT00939575 | Pre-eclampsia and Metabolomics (GEM-1) | unknown status |
| ClinicalTrials.gov ID: NCT06319014 | Physical Activity to Mitigate PreEclampsia Risk (PAMPER) | recruiting |
| ClinicalTrials.gov ID: NCT05445544 | A Multicenter Cohort Study on Maternal and Infant Microecology and New Targets for Pre-eclampsia Screening in China (CALM2001) | recruiting |
| ClinicalTrials.gov ID: NCT06430398 | A Novel Multiomic AI Approach for Early Preeclampsia Prediction in Pregnancy (PREMEM) | recruiting |
| ClinicalTrials.gov ID: NCT01891240 | IMproved PRegnancy Outcome by Early Detection (IMPROvED) | unknown status |
| ClinicalTrials.gov ID NCT06421493 | Role of the Maternal Microbiota on the Immune Response and Metabolism During Hypertensive Disorders (MATER) | recruiting |
