# Supplementary material for: Untargeted metabolomics for the early detection of preeclampsia: A systematic review of human studies
Source: PLoS One. 2026 Mar 30;21(3):e0339292. doi: 10.1371/journal.pone.0339292 (PMC13035155; doi:10.1371/journal.pone.0339292)
Supplement: S3 Table — (DOCX) [file pone.0339292.s006.docx]

**S6 Table. Excluded studies at fulltext screening**

| **Author** | **Title** | **Principal Reason for exclusion** |
| --- | --- | --- |
| Diboun, I. et al. 2020 | Metabolic profiling of pre-gestational and gestational diabetes mellitus identifies novel predictors of pre-term delivery. | wrong outcome |
| Huhtala, M.S. et al. 2018 | Amino acid profile in women with gestational diabetes mellitus treated with metformin or insulin. | wrong outcome |
| Zhang, Y. et al. 2017 | Maternal low thyroxin levels are associated with adverse pregnancy outcomes in a Chinese population. | wrong outcome |
| Polsani, S. et al. 2013 | Emerging new biomarkers of preeclampsia. | wrong outcome |
| Mayrink, J. et al. 2022 | Prediction of pregnancy-related hypertensive disorders using metabolomics: a systematic review. | wrong outcome |
| Burton, G. et al. 2019 | Pre-eclampsia: pathophysiology and clinical implications. | wrong outcome |
| Kawasaki, K. et al. 2019 | Metabolomic Profiles of Placenta in Preeclampsia. | wrong intervention |
| Zhou, X. et al. 2017 | Impaired mitochondrial fusion, autophagy, biogenesis and dysregulated lipid metabolism is associated with preeclampsia. | wrong outcome |
| Stewart, Z.A. et al. 2021 | Reliance on lipid and protein energy sources is associated with materno-fetal complications in type 1 diabetes pregnancy: a CONCEPTT trial substudy | wrong outcome |
| May, L. et al. 2024 (last update) | Pregnancy Exercise Mode Effect on Childhood Obesity | not published results |
| Birchenall, K. et al. 2024 (last update) | The PIONEER Study - A study to investigate whether taking the medication Pravastatin reduces the number of babies born too early (preterm, i.e., before 37 weeks of pregnancy) and, if so, how it works in the body to do this | not published results |
| Hartmann, S. et al. 2023 | Can single-cell and spatial omics unravel the pathophysiology of pre-eclampsia? | wrong outcome |
| McArthur, KL. et al. 2022 | Trimethylamine N-Oxide and Its Precursors Are Associated with Gestational Diabetes Mellitus and Pre-Eclampsia in the Boston Birth Cohort | wrong outcome |
| Mayrink, J. et al. 2020 | Metabolomics for prediction of hypertension in pregnancy: a systematic review and meta-analysis protocol | wrong study design |
| MacDonald, M.T. et al. 2022 | Clinical tools and biomarkers to predict preeclampsia | wrong study design |
| McKeating, DR. et al. 2019 | Elemental Metabolomics and Pregnancy Outcomes | wrong outcome |
| McBride, N. et al. 2021 | Do Mass Spectrometry-Derived Metabolomics Improve the Prediction of Pregnancy-Related Disorders? Findings from a UK Birth Cohort with Independent Validation | wrong outcome |
| Dunn, W.B. et al. 2012 | The metabolome of human placental tissue: investigation of first trimester tissue and changes related to preeclampsia in late pregnancy | wrong population |
| Kelly, R.S. et al. 2017 | Integration of metabolomic and transcriptomic networks in pregnant women reveals biological pathways and predictive signatures associated with preeclampsia | wrong outcome |
| McBride, N. et al. 2020 | Do nuclear magnetic resonance (NMR)-based metabolomics improve the prediction of pregnancy-related disorders? Findings from a UK birth cohort with independent validation | wrong outcome |
| Yang, XT. et al. 2018 | AMPK Hyper-Activation Alters Fatty Acids Metabolism and Impairs Invasiveness of Trophoblasts in Preeclampsia | wrong outcome |
| Heazell, A.E.P. et al. 2012 | A Metabolomic Approach Identifies Differences in Maternal Serum in Third Trimester Pregnancies That End in Poor Perinatal Outcome | wrong outcome |
| Burnum-Johnson, KE. et al. 2017 | Characterizing the lipid and metabolite changes associated with placental function and pregnancy complications using ion mobility spectrometry-mass spectrometry and mass spectrometry imaging | wrong outcome |
| Virgiliou, C. et al. 2017 | Amniotic Fluid and Maternal Serum Metabolic Signatures in the Second Trimester Associated with Preterm Delivery | wrong outcome |
| Weingrill, R.B. et al. 2021 | Exosome-Enriched Plasma Analysis as a Tool for the Early Detection of Hypertensive Gestations | wrong outcome |
| McKeating, D.R. et al. 2020 | Elemental metabolomics in human cord blood: Method validation and trace element quantification | wrong outcome |
| Ferguson, K. et al. 2017 | Repeated measures of inflammation and oxidative stress biomarkers in preeclamptic and normotensive pregnancies | wrong outcome |
| Nobakht M. Gh, B. F. 2018 | Application of metabolomics to preeclampsia diagnosis | wrong study design |
| Yao, M. et al. 2022 | Identification of Biomarkers for Preeclampsia Based on Metabolomics | wrong study design |
| Fanos, V. et al. 2023 | Metabolomics Application in Maternal-Fetal Medicine | wrong study design |
| Liu, X.-F. et al. 2023 | Magnetic resonance spectroscopy and liquid chromatography-mass spectrometry metabolomics study may differentiate pre-eclampsia from gestational hypertension | wrong population |
