# Supplementary material for: Untargeted metabolomics for the early detection of preeclampsia: A systematic review of human studies
Source: PLoS One. 2026 Mar 30;21(3):e0339292. doi: 10.1371/journal.pone.0339292 (PMC13035155; doi:10.1371/journal.pone.0339292)
Supplement: S3 Appendix — (DOCX) [file pone.0339292.s007.docx]

| **Section and Topic** | **Item #** | **Checklist item** | **Location where item is reported** |
| --- | --- | --- | --- |
| **TITLE** | | |  |
| Title | 1 | Identify the report as a systematic review. | Page 1 |
| **ABSTRACT** | | |  |
| Abstract | 2 | See the PRISMA 2020 for Abstracts checklist. | Page 1 |
| **INTRODUCTION** | | |  |
| Rationale | 3 | Describe the rationale for the review in the context of existing knowledge. | Page 2-3 |
| Objectives | 4 | Provide an explicit statement of the objective(s) or question(s) the review addresses. | Page 4 |
| **METHODS** | | |  |
| Eligibility criteria | 5 | Specify the inclusion and exclusion criteria for the review and how studies were grouped for the syntheses. | Page 5 |
| Information sources | 6 | Specify all databases, registers, websites, organisations, reference lists and other sources searched or consulted to identify studies. Specify the date when each source was last searched or consulted. | Page 4 |
| Search strategy | 7 | Present the full search strategies for all databases, registers and websites, including any filters and limits used. | Page 4-5 |
| Selection process | 8 | Specify the methods used to decide whether a study met the inclusion criteria of the review, including how many reviewers screened each record and each report retrieved, whether they worked independently, and if applicable, details of automation tools used in the process. | Page 4-6 |
| Data collection process | 9 | Specify the methods used to collect data from reports, including how many reviewers collected data from each report, whether they worked independently, any processes for obtaining or confirming data from study investigators, and if applicable, details of automation tools used in the process. | Page 4-6 |
| Data items | 10a | List and define all outcomes for which data were sought. Specify whether all results that were compatible with each outcome domain in each study were sought (e.g. for all measures, time points, analyses), and if not, the methods used to decide which results to collect. | Page 4-6 |
|  | 10b | List and define all other variables for which data were sought (e.g. participant and intervention characteristics, funding sources). Describe any assumptions made about any missing or unclear information. | Page 4-6, no assumptions were made |
| Study risk of bias assessment | 11 | Specify the methods used to assess risk of bias in the included studies, including details of the tool(s) used, how many reviewers assessed each study and whether they worked independently, and if applicable, details of automation tools used in the process. | Page 6, Fig S4 |
| Effect measures | 12 | Specify for each outcome the effect measure(s) (e.g. risk ratio, mean difference) used in the synthesis or presentation of results. | No quantitative synthesis or meta-analysis was performed. Therefore, no effect measures were calculated. The results were summarized narratively |
| Synthesis methods | 13a | Describe the processes used to decide which studies were eligible for each synthesis (e.g. tabulating the study intervention characteristics and comparing against the planned groups for each synthesis (item #5)). | Page 5 |
|  | 13b | Describe any methods required to prepare the data for presentation or synthesis, such as handling of missing summary statistics, or data conversions. | As no meta-analysis was performed, no statistical transformations were required. Data were extracted and presented as reported in the original studies. Studies were grouped narratively according to the biological matrix analyzed |
|  | 13c | Describe any methods used to tabulate or visually display results of individual studies and syntheses. | Summary tables (table 1 and 2) |
|  | 13d | Describe any methods used to synthesize results and provide a rationale for the choice(s). If meta-analysis was performed, describe the model(s), method(s) to identify the presence and extent of statistical heterogeneity, and software package(s) used. | No meta-analysis was conducted |
|  | 13e | Describe any methods used to explore possible causes of heterogeneity among study results (e.g. subgroup analysis, meta-regression). | Not performed. Due to the small number of included studies and substantial clinical and methodological heterogeneity, no formal exploration was feasible |
|  | 13f | Describe any sensitivity analyses conducted to assess robustness of the synthesized results. | Not conducted. No quantitative synthesis was performed, and the limited number and heterogeneity of included studies precluded meaningful sensitivity analyses. |
| Reporting bias assessment | 14 | Describe any methods used to assess risk of bias due to missing results in a synthesis (arising from reporting biases). | ClinicalTrials.gov was searched to identify completed but unpublished studies related to metabolomics and preeclampsia, aiming to assess potential reporting bias. No completed studies meeting the inclusion criteria were found, suggesting minimal risk of bias due to missing results |
| Certainty assessment | 15 | Describe any methods used to assess certainty (or confidence) in the body of evidence for an outcome. | No formal assessment of certainty or confidence in the body of evidence was performed. However, risk of bias was assessed for all included studies |
| **RESULTS** | | |  |
| Study selection | 16a | Describe the results of the search and selection process, from the number of records identified in the search to the number of studies included in the review, ideally using a flow diagram. | Page 6, Figure 1 |
|  | 16b | Cite studies that might appear to meet the inclusion criteria, but which were excluded, and explain why they were excluded. | Not reported |
| Study characteristics | 17 | Cite each included study and present its characteristics. | Page 7-8 and Table 1 |
| Risk of bias in studies | 18 | Present assessments of risk of bias for each included study. | Figure S4 |
| Results of individual studies | 19 | For all outcomes, present, for each study: (a) summary statistics for each group (where appropriate) and (b) an effect estimate and its precision (e.g. confidence/credible interval), ideally using structured tables or plots. | Summary statistics and key findings from each study were presented narratively and in structured tables. No effect estimates or confidence intervals were synthesized, as no meta-analysis was conducted |
| Results of syntheses | 20a | For each synthesis, briefly summarise the characteristics and risk of bias among contributing studies. | Study characteristics and risk of bias were summarized in Table 1 and Figure S4 respectively |
|  | 20b | Present results of all statistical syntheses conducted. If meta-analysis was done, present for each the summary estimate and its precision (e.g. confidence/credible interval) and measures of statistical heterogeneity. If comparing groups, describe the direction of the effect. | No statistical synthesis or meta-analysis was performed due to heterogeneity in study designs, outcomes, and analytical methods |
|  | 20c | Present results of all investigations of possible causes of heterogeneity among study results. | No investigation of heterogeneity was conducted. However, heterogeneity was qualitatively explored by grouping studies according to biological matrix and analytical platform |
|  | 20d | Present results of all sensitivity analyses conducted to assess the robustness of the synthesized results. | No sensitivity analyses were conducted, as no quantitative synthesis was performed and the limited number and heterogeneity of included studies precluded such analyses |
| Reporting biases | 21 | Present assessments of risk of bias due to missing results (arising from reporting biases) for each synthesis assessed. | No assessment of reporting bias was conducted. However, ClinicalTrials.gov was searched to identify completed but unpublished studies. No eligible unpublished studies were found, suggesting low concern for reporting bias |
| Certainty of evidence | 22 | Present assessments of certainty (or confidence) in the body of evidence for each outcome assessed. | No formal assessment of certainty or confidence in the body of evidence was performed. However, risk of bias was assessed for all included studies |
| **DISCUSSION** | | |  |
| Discussion | 23a | Provide a general interpretation of the results in the context of other evidence. | Page 22-26 |
|  | 23b | Discuss any limitations of the evidence included in the review. | Page 22 and 25 |
|  | 23c | Discuss any limitations of the review processes used. | Page 25 |
|  | 23d | Discuss implications of the results for practice, policy, and future research. | Page 25-26 |
| **OTHER INFORMATION** | | |  |
| Registration and protocol | 24a | Provide registration information for the review, including register name and registration number, or state that the review was not registered. | Page 1 and 4 |
|  | 24b | Indicate where the review protocol can be accessed, or state that a protocol was not prepared. | No formal protocol was prepared for this review |
|  | 24c | Describe and explain any amendments to information provided at registration or in the protocol. | No amendments were made to the protocol after registration |
| Support | 25 | Describe sources of financial or non-financial support for the review, and the role of the funders or sponsors in the review. | Not reported |
| Competing interests | 26 | Declare any competing interests of review authors. | The authors have previously declared any competing interests during PROSPERO registration |
| Availability of data, code and other materials | 27 | Report which of the following are publicly available and where they can be found: template data collection forms; data extracted from included studies; data used for all analyses; analytic code; any other materials used in the review. | Reported in the review protocol |

*From:*  Page MJ, McKenzie JE, Bossuyt PM, Boutron I, Hoffmann TC, Mulrow CD, et al. The PRISMA 2020 statement: an updated guideline for reporting systematic reviews. BMJ 2021;372:n71. doi: 10.1136/bmj.n71
