# Supplementary material for: Untargeted metabolomics for the early detection of preeclampsia: A systematic review of human studies
Source: PLoS One. 2026 Mar 30;21(3):e0339292. doi: 10.1371/journal.pone.0339292 (PMC13035155; doi:10.1371/journal.pone.0339292)
Supplement: Attachment — Submitted filename: Response_To_Reviewers.docx [file pone.0339292.s009.docx]

Research Centre CIAIMBITAL

Department of Chemistry and Physics

Universidad de Almería, E-04120 Almería

Telephone:	+34 950 214 465

E-mail:	[ifernan@ual.es](mailto:ifernan@ual.es)

Web:	www.nmrmbc.com

List of Changes

Wednesday, January 21, 2026

Prof. María Teresa Llinás

Academic Editor PLOS One

Dear Editor,

We are pleased that both reviewers have recommended the publication of our manuscript after major revisions. We have revised the manuscript as requested, addressing all the concerns detailed below. Additionally, we are uploading a file with all the changes highlighted in yellow.

**Reviewer #1**

1) Although the authors state that a metaanalysis is not possible due to differing methods between the studies, an extended analysis on the clinical utility of metabolomics in preeclampsia is warranted, especially limited to MS or NMR metabolomics.

**RESPONSE TO THE REVIEWER:** We have added a discussion on the clinical utility of metabolomics in preeclampsia, focusing on MS and NMR techniques. We explain how these methods could help with early risk assessment, disease stratification, and monitoring of PE, while noting limitations due to differences in study design, gestational age, PE subtype, and analytical methods. Examples from other diseases, such as breast cancer and multiple sclerosis, are mentioned now to show the potential of these techniques in clinical research.

2) Figure 2 attempts to summarise findings on the same metabolites between studies, but ignore differences in direction of change between studies. Would it be possible to include the direction of change, maybe as a color code? The figure also conflates the different study matrices like placenta and serum/plasma. It would be nice to see in the figure which matrix is discussed when referring to a metabolite.

**RESPONSE TO THE REVIEWER:** Done. Figure 2 has been ammended, and now both matrices and direction of change are included as color codes.

3) Similarly, when describing each study, a note of timing of pregnancy would benefit interpretability.

**RESPONSE TO THE REVIEWER:** We have now included the timing of pregnancy for all studies in which this information was not previously cited in the text.

4) Authors cite differing metabolic states of patients as a reason for the differences between studies, but do not discuss methodological and preprocessing differences between studies, for example normalization strategies and quantification methods. The review would benefit from a short discussion on these limitations as well.

**RESPONSE TO THE REVIEWER:** We have added a short discussion highlighting that methodological and data preprocessing differences across studies may contribute to variability in reported metabolomic findings.

5) Given the heterogeneity of findings in the studies, what is a way forward for the field. Validation of previous findings, larger exploratory studies, more specific and reproducible methodology? please discuss

**RESPONSE TO THE REVIEWER:** We agree with the reviewer that the heterogeneity of findings across studies represents a major challenge for the field. We have now expanded the Discussion section to explicitly address possible ways forward. In particular, we emphasize the need for: (i) stratification of study cohorts according to preeclampsia subtypes (e.g., early-onset vs. late-onset PE), (ii) validation of previously reported biomarkers in independent and adequately powered cohorts, and (iii) harmonization of pre-analytical procedures, sample collection protocols, and analytical workflows to improve reproducibility across studies. We also discuss the importance of combining exploratory metabolomic studies with targeted, hypothesis-driven approaches to enhance the translational and clinical relevance of metabolomic findings in preeclampsia.

6) Line 57, EO and LO PE are described as “usually occurring”, “normally presents” before/after 34 weeks. This is the actual definition of EO/LO PE so there is no usuall/normally, these always occur in their defined GA periods.

**RESPONSE TO THE REVIEWER:** Done. These expressions have been removed from the text.

7) Preeclampsia is written in full several times even though an abbreviation is provided at the start. Examples: PE defined line 48, preeclampsia spelled out line line 75, 102, 222,340 etc.

**RESPONSE TO THE REVIEWER:** Done. “PE” is now used consistently throughout the manuscript.

8) Weeks of gestation defined on line 340 as wkGA but then not used again, we consider appropriate to retain it in the manuscript.

**RESPONSE TO THE REVIEWER:** The abbreviation wkGA is indeed used later in the manuscript (lines 343–344) to refer to weeks of gestation. Therefore, we consider it appropriate to retain this abbreviation in the manuscript for consistency and clarity.

9) Gestational hypertension defined as GHT on line 258 (not actually defined in the paper) and as GH on line 267.

**RESPONSE TO THE REVIEWER:** Gestational hypertension has been defined now in the introduction as GH. Other abbreviations referring to this condition have been changed to GH.

10) Pregnant controls are inconsistently defined as PC (357), “Healthy controls”(394), “normotensive pregnancies”, “normal pregnancies”, “healthy women”. Although different nomenclature might reflect different populations, the review would improve readability with consistent naming of non-pregnant women, pregnant women without HDP, and PE/GH/HDP populations.

**RESPONSE TO THE REVIEWER:** We have reviewed all terminology to ensure it accurately reflects the study populations. Non-pregnant women are referred to as NP, and pregnant women without hypertensive disorders of pregnancy are referred to as pregnant controls (PC). In the single case where the original article defined PC differently (not as women without HDP), we have retained the terminology from the original study to accurately reflect that population. Affected groups are designated as PE, GH, or HDP, as appropriate. All abbreviations are defined at first mention and applied uniformly across the manuscript, tables, and figures.

11) PLSDA defined several times: (221,255, 293). PCA mentioned at line 377, but not at first mention (328).

**RESPONSE TO THE REVIEWER:** Done. Only one definition of PLS-DA has been retained, with the repeated definitions removed, and the definition of PCA has been added at its first mention.

12) Some metabolites are inconsistently named: L-glutamine, L-histidine (362, 359), L-valine (390) valine (439) glutamine, histidine (374). Similarly, please refer to metabolites by a consistent name: citrate (367) is the same metabolite as ascorbate (416). Accordingly, does this change your analysis if you did not realize they were the same?

**RESPONSE TO THE REVIEWER:** Done. We thank the reviewer for pointing out the inconsistent metabolite nomenclature. We have revised the manuscript to ensure consistent naming throughout (L-glutamine, L-histidine and L-valine are now used uniformly). Regarding citrate and ascorbate, we clarify that these are chemically and biologically distinct metabolites derived from citric acid and ascorbic acid, respectively. They were therefore intentionally treated as separate metabolites in our analysis. This clarification does not affect the analysis or the interpretation of the results.

13) Authors refer to results from logistic regression studies both as AUC (area under curve) (213, 243, 272) and ROC curve area (315, 330), please stay consistent.

**RESPONSE TO THE REVIEWER:** Done. We agree that consistent terminology is important. Accordingly, all instances previously referred to as “ROC” have now been updated to AUC throughout the manuscript.

14) FGR not defined (line 432)

**RESPONSE TO THE REVIEWER:** Done. Definition included.

15) Authors suggest that differences in metabolites between studies might be due to metabolic profiles of the patients (458), this is a circular argument: differences in metabolites are due to differences in metabolites. Please refine your argument

**RESPONSE TO THE REVIEWER:** We agree that the original statement was circular and did not sufficiently advance the interpretation of inter-study variability. Accordingly, this argument has been removed, and the Discussion has been restructured to focus instead on well-defined sources of heterogeneity, including differences in study design, patient stratification, clinical characteristics, and analytical methodologies.

16) Wording on line 70 is confusing, please rephrase “PlGF just can predict less 90% of early PE cases”. Also do you mean EO-PE as defined earlier?

**RESPONSE TO THE REVIEWER:** The wording on line 70 has been re`pphrased as sugested. It now correctly indicates that PlGF predicts less than 90% of early-onset PE (EO-PE) cases, as previously defined. The reference has also been corrected, as the original one was incorrect.

17) Paragraph on line 84 is not very relevant, describing cancer and Covid-19. It can be shortened to a few sentences. Perhaps a better use of this space is to provide an overview of other more or less validated biomarkers of preeclampsia, for example sFLT, PlGF and PAPP-A? This would help when these are discussed later in line 338.

**RESPONSE TO THE REVIEWER:** The paragraph on line 84 has been shortened as suggested, and an overview of other commonly used biomarkers for preeclampsia, including sFlt-1, PlGF, and PAPP-A, has been added. For clarity and flow, this information has been included in line 67.

18) Table 1, column “Age, average years” is unclear. What do the two numbers mean in row 1,3,6 etc. Only last row has a measure of confidence, are these standard deviance?

**RESPONSE TO THE REVIEWER:** All data in that column has been corrected. Now it is presented as “Maternal age, (mean ± SD or median [range])”, providing a clear measure.

19) Line 221, extra period after citation

**RESPONSE TO THE REVIEWER:** Done.

20) Uterine doppler PI mentioned line 246 but defined with abbreviation at line 383

**RESPONSE TO THE REVIEWER:** Done. For clarity and consistency, we have standardized the terminology throughout the manuscript to uterine artery pulsatility index (UtAPI), and the abbreviation has been defined at its first mention.

21) Line 273 refers to an AUC of 0, is this correct?

**RESPONSE TO THE REVIEWER:** No, this was not correct. The AUC value is 0.177, and this has been corrected in the text.

22) Throughout the description of the included studies, a lot of metabolites are listed. These are repeated in the table 2. Would it be possible to reduce the mentioned metabolites in text to a few most interesting ones, to improve readability? For example lines 303-310.

**RESPONSE TO THE REVIEWER:** We have now highlighted the most representative metabolite classes (fatty acids, acylcarnitines, and amino acid–related compounds) and all individual metabolites remain available in Table 3.

23) Table 2, abbreviations not defined in table heading/footer.

**RESPONSE TO THE REVIEWER:** The corresponding definitions for all abbreviations have now been added to the footnote of the tables.

24) Seems unnecessary to define abbreviations that are not used again, lines 296-299.

**RESPONSE TO THE REVIEWER:** The abbreviations have been removed, as the article that mentioned them has been excluded from the study.

25) Line 408, does this refer to healthy pregnant women or non pregnant women?

**RESPONSE TO THE REVIEWER:** It refers to healthy pregnant women, it has been changed in the text.

26) Line 430, unclear grammar. Should it be “contains almost all”?

**RESPONSE TO THE REVIEWER:** The sentence has been revised for clarity, and it now reads “which contained almost all LO-PE pregnancies…”.

**Reviewer #2**

1) The PICO question is not fully adhered to in the final study selection. The article by Liu et al. (reference 30) does not compare the group of women with preeclampsia to women with healthy pregnancies. The authors should either exclude it from the review or justify its inclusion in the text and explain the implications of this choice.

**RESPONSE TO THE REVIEWER:** Although the study by Liu et al. (reference 30) included women with healthy pregnancies, the metabolomic comparisons were primarily performed between women with preeclampsia and those with gestational hypertension, rather than between preeclampsia and normotensive pregnancies. As this does not strictly adhere to the predefined PICO criteria of the review, the study has now been excluded from the final analysis. It has also been removed from the corresponding tables and figures and is now listed in the appendix of excluded studies.

2) The authors do not conduct any subgroup analyses despite the evident heterogeneity among the included studies. Instead, they restrict their approach to presenting individual descriptions of each study. Accordingly, the authors should consider the following: Several studies pool early-onset PE with late-onset PE, whereas others, such as Bahado-Shing et al. (reference 24), focus exclusively on early-onset PE. This increases the heterogeneity of the review and makes it difficult to interpret the results as a whole. The authors should explicitly address this issue in both the analysis of the results and the discussion.

**RESPONSE TO THE REVIEWER:** The type of preeclampsia evaluated in each study (early-onset, late-onset, mixed, or not specified) is now reported in the Participants Characteristics section and has been included in Table 2. In addition, we have added a sentence in the Results section explicitly acknowledging this source of clinical heterogeneity. This issue is further discussed in the Discussion section.

3) The data synthesis also does not distinguish between study design, biological matrices, analytical methodology, or timing of sample collection. The review combines all these dimensions without a sufficiently differentiated structure, which weakens the conclusions. If the aim of the review is to evaluate the use of metabolomics for the prediction of preeclampsia, it is critical to consider the gestational trimester in which measurements are taken. Placental studies are conducted postpartum, and therefore contribute limited information on the predictive value of metabolomics. Early predictive biomarkers are mixed with late diagnostic biomarkers, making it difficult to draw any firm conclusions about the early predictive value of the reported metabolites.

**RESPONSE TO THE REVIEWER:** Study design, biological matrices, analytical methodology, and timing of sample collection are clearly reported in the Results tables. Additionally, the results are structured by biological matrix, and for each study, the analytical technique and sample collection timing are now explicitly indicated. Studies conducted in placental tissue have been considered in the Discussion, and we have added a clarifying sentence noting that the metabolites identified in placenta largely reflect the metabolic state at delivery and are therefore descriptive of PE at that time. Importantly, some of these metabolites were also detected in maternal serum and plasma during pregnancy, suggesting that they may have potential as early predictive biomarkers.

4) The manuscript concludes that there is convergence in four metabolites (alanine, lactate, glutamate, glutamine), yet these metabolites are reported as increased in some contexts and decreased in others. The authors should provide a more cautious narrative synthesis of these common metabolites and clearly indicate when each metabolite is increased, decreased, or unchanged according to sample type and analytical technique.

**RESPONSE TO THE REVIEWER:** The direction of change for each metabolite (whether increased or decreased) is now clearly indicated in all instances, with explicit mention of the sample type. In addition, we have now added the analytical technique used for each measurement to provide full context. Furthermore, Figure 2 has been updated to reflect these changes. It now not only summarizes the metabolites but also indicates whether their levels increase or decrease and specifies the sample matrix analyzed.

5) Explaining the methodological heterogeneity between metabolomic technologies is essential. The review does not discuss how he different techniques (NMR, LC–MS, HR-MAS, etc) may yield different metabolite profiles. The level of confidence with which each study identifies its metabolites should be rigorously evaluated. This would help distinguish high-confidence signals from those that may correspond to isomers or analytical artefacts and would prevent treating as common metabolites those findings that might instead reflect methodological or analytical limitations.

**RESPONSE TO THE REVIEWER:** We have substantially expanded the Study Characteristics section to include a dedicated analysis of the metabolomic platforms used across the selected studies, explicitly discussing their analytical principles, strengths, and limitations. This comparison is summarized in a new table (Table 1). In addition, we have added a specific paragraph to the Discussion addressing how methodological heterogeneity across platforms (NMR, LC–MS, LC–MS/MS, HR-MAS, etc.) influences metabolite coverage, sensitivity, and, critically, the confidence of metabolite identification. We now explicitly discuss issues related to isomer discrimination, matrix effects, and analytical artefacts, and emphasize that metabolites consistently reported across multiple studies and platforms are more likely to represent high-confidence biological signals, whereas technique-specific findings may partly reflect methodological or analytical constraints rather than true biological variation.

We hope that all these changes will be satisfactory, and we are uploading the revised versions of manuscript and supporting information. We are confident that the changes done will meet with your approval.

Many thanks in advance.

Sincerely,

Ignacio Fernández
