# Supplementary material for: Untargeted metabolomics for the early detection of preeclampsia: A systematic review of human studies
Source: PLoS One. 2026 Mar 30;21(3):e0339292. doi: 10.1371/journal.pone.0339292 (PMC13035155; doi:10.1371/journal.pone.0339292)
Supplement: Attachment — Submitted filename: Response to Reviewers.docx [file pone.0339292.s010.docx]

Research Centre CIAIMBITAL

Department of Chemistry and Physics

Universidad de Almería, E-04120 Almería

Telephone:	+34 950 214 465

E-mail:	[ifernan@ual.es](mailto:ifernan@ual.es)

Web:	www.nmrmbc.com

List of Changes

Sunday, March 1, 2026

Prof. María Teresa Llinás

Academic Editor PLOS One

Dear Editor,

We have revised the manuscript as requested, addressing all the concerns detailed below. Additionally, we are uploading a file with all the changes highlighted in red.

**Reviewer #2**

We sincerely thank the reviewer for the careful and constructive evaluation of our manuscript.

1) First, the interpretation of alanine discrepancies remains overly generic. Attributing opposite directions of change to “analytical techniques” and “clinical heterogeneity” is insufficient without a more explicit mechanistic rationale. The discussion should distinguish between systemic versus placental metabolism and between early adaptive metabolic shifts and later-stage dysfunction. Without this clarification, the explanatory model remains incomplete. Moreover, this limitation also weakens the strength of the overall conclusions of the review, as the proposed convergence of metabolites cannot be adequately supported if the direction and underlying biological context of these changes are not coherently interpreted.

**RESPONSE TO THE REVIEWER:** We agree with the reviewer that temporal stage and biological compartment are essential for interpreting metabolomic findings in preeclampsia. To address this, we have added a new opening paragraph to the Discussion explicitly introducing a framework that distinguishes: (i) early predictive maternal signals vs. late descriptive placental changes, and (ii) systemic maternal metabolism vs. local placental metabolism. This paragraph clarifies that apparent inconsistencies in metabolite directionality are expected when observations derive from different physiological phases or compartments rather than representing purely analytical discrepancies. This conceptual framing now guides the interpretation of subsequent metabolite-specific sections.

Regarding the interpretation of alanine discrepancies, we have substantially rewritten the alanine section. The revised text now: i) distinguishes maternal systemic metabolism from placental metabolism; ii) separates early adaptive metabolic responses from late-stage dysfunction; and iii) proposes a mechanistic interpretation linking alanine dynamics to altered hepatic gluconeogenesis, nitrogen transport, and systemic metabolic adaptation in early pregnancy. This restructuring provides a physiologically grounded explanation for opposite directions of change across studies and matrices.

2) Second, the explanation of lactate findings remains physiologically inconsistent. The reported decrease in lactate was observed in first trimester samples from women who subsequently developed late-onset PE. Nevertheless, the discussion relies on placental hypoxia as the main mechanistic explanation. Placental hypoxia is typically associated with later stage or early onset PE rather than first trimester preclinical LO-PE, and hypoxia-driven anaerobic glycolysis would be expected to increase lactate production, not decrease it. As currently written, the proposed mechanism does not adequately account for the observed direction of change. The authors should resolve this discrepancy and provide a pathophysiological explanation that is consistent with the specific gestational window studied rather than extrapolating mechanisms from later stages of disease.

**RESPONSE TO THE REVIEWER:** We agree that attributing first-trimester lactate reductions to placental hypoxia was not appropriate. The lactate section has therefore been revised to distinguish early systemic metabolic adaptation from later hypoxia-driven placental dysfunction. The revised discussion now proposes that reduced early lactate may reflect altered maternal metabolic flexibility or gluconeogenic regulation rather than hypoxia, which is more relevant to established disease stages. This modification aligns the mechanistic interpretation with the gestational window studied.

3) With respect to the section of the Discussion addressing glutamate and glutamine, clearer compartmental and temporal differentiation is needed. Serum and placental findings are presented within a unified framework without sufficiently distinguishing systemic maternal metabolism from local placental processes. I would recommend restructuring this section to clearly distinguish systemic maternal findings from placental tissue results and to integrate a temporal perspective (early predictive vs late descriptive changes).

**RESPONSE TO THE REVIEWER:** We have restructured this section to explicitly separate maternal biofluid findings from placental tissue observations and to distinguish predictive signals from descriptive late-stage metabolic alterations. The revised text now integrates these metabolites within systemic redox balance, immune regulation, and nitrogen metabolism, while clearly acknowledging the temporal context of each observation.

4) Finally, the concluding statement suggesting that the current evidence provides a “solid basis” for future validation appears overly optimistic, given the limited number of studies, the inconsistency in the direction of metabolite changes, and the absence of quantitative synthesis. A more cautious and proportionate tone would be advisable to better reflect the strength of the available evidence.

**RESPONSE TO THE REVIEWER:** We agree and have moderated the tone of the concluding paragraph. The revised conclusion now explicitly acknowledges the limited number of studies, variability in gestational timing, PE subtype definitions, and analytical platforms, as well as the inconsistent directionality observed for some metabolites. Rather than presenting metabolomics as providing definitive predictive biomarkers, the revised text emphasizes that the current literature primarily identifies plausible metabolic pathways and physiological axes that warrant further investigation. This change ensures that the conclusions remain proportionate to the strength of the available evidence.

We hope that all these changes will be satisfactory, and we are uploading the revised version of the manuscript We are confident that the changes done will meet with your approval.

Many thanks in advance.

Sincerely,

Ignacio Fernández
