# Supplementary material for: Untargeted metabolomics for the early detection of preeclampsia: A systematic review of human studies
Source: PLoS One. 2026 Mar 30;21(3):e0339292. doi: 10.1371/journal.pone.0339292 (PMC13035155; doi:10.1371/journal.pone.0339292)
Supplement: Attachment — Submitted filename: Response_to_Reviewers_auresp_3.docx [file pone.0339292.s011.docx]

Research Centre CIAIMBITAL

Department of Chemistry and Physics

Universidad de Almería, E-04120 Almería

Telephone:	+34 950 214 465

E-mail:	[ifernan@ual.es](mailto:ifernan@ual.es)

Web:	www.nmrmbc.com

List of Changes

Monday, March 9, 2026

Prof. María Teresa Llinás

Academic Editor PLOS One

Dear Editor,

We have revised the manuscript as requested, addressing all the concerns detailed below. Additionally, we are uploading a file with all the changes highlighted in red.

**Reviewer #2**

We sincerely thank the reviewer for the careful evaluation of our manuscript and for the constructive suggestions that have helped us improve the internal coherence and interpretative strength of the Discussion section. Below we address each point in detail.

1) The interpretation of alanine and lactate would benefit from a more integrated explanation within the pyruvate metabolic framework… The study discussed reports decreased levels of both metabolites in first-trimester maternal samples… consider alternative explanations…

**RESPONSE TO THE REVIEWER:** We have clarified that the hypoxia-driven metabolic routing described is more characteristic of later stages of established disease rather than the early gestational window captured in first-trimester maternal metabolomic studies. In addition, we have incorporated alternative physiological explanations for the simultaneous decrease in lactate and alanine, including increased hepatic utilization of both metabolites as gluconeogenic substrates, enhanced mitochondrial oxidative use of pyruvate, and metabolic heterogeneity across PE subtypes. These additions reconciles apparently divergent findings and improve the internal consistency of the Discussion.

2) The discussion of glutamate and glutamine could be more explicitly integrated with the previously described pyruvate–alanine–lactate metabolic axis.

**RESPONSE TO THE REVIEWER:** We agree and have revised the text to present glutamate and glutamine metabolism within an integrated metabolic network. As suggested, we now emphasize how transamination reactions functionally connect amino acid metabolism with central carbon metabolism, linking the glutamate–glutamine axis to the pyruvate–alanine–lactate pathway.

3) The Discussion could further emphasize the interpretative contribution of the review… provide a coherent synthesis…

**RESPONSE TO THE REVIEWER:** We appreciate this important suggestion. To strengthen the integrative perspective, we have revised the concluding section to emphasize the interpretative value of the review. The revised text highlights how apparently distinct metabolite alterations converge into interconnected metabolic networks and coherent physiological axes, reinforcing the added value of the review beyond summarizing individual studies.

4) Two paragraphs convey very similar ideas regarding compartment- and stage-dependent metabolic differences… Streamlining or merging would help reduce redundancy…

**RESPONSE TO THE REVIEWER:** We agree and have streamlined the Discussion accordingly. The earlier general paragraph describing compartment- and stage-dependent differences has been retained, while the later paragraph has been condensed to avoid conceptual redundancy and improve clarity and flow.

We thank the reviewer again for these valuable suggestions, and we hope that all these changes will be satisfactory. We are uploading the revised version of the manuscript and are confident that the changes done will meet with your approval.

Many thanks in advance.

Sincerely,

Ignacio Fernández
